# Distribution and viability of spermatozoa in the canine female genital tract during post-ovulatory oocyte maturation

**DOI:** 10.1186/1751-0147-54-49

**Published:** 2012-08-29

**Authors:** Inga Karre, Andrea Meyer-Lindenberg, Carola Urhausen, Andreas Beineke, Burkhard Meinecke, Marion Piechotta, Martin Beyerbach, Anne-Rose Günzel-Apel

**Affiliations:** 1 Small Animal Clinic, Veterinary University Hannover, Foundation, Bünteweg 15, 30559, Hannover, Germany; 2 Department of Pathology, Veterinary University Hannover, Foundation, Bünteweg 17, 30559, Hannover, Germany; 3 Department of Reproductive Biology, Veterinary University Hannover, Foundation, Bünteweg 2, 30559, Hannover, Germany; 4 Clinic for Cattle, Veterinary University Hannover, Foundation, Bischofsholer Damm 15, 30173, Hannover, Germany; 5 Department of Biometry, Epidemiology and Information Processing, Veterinary University Hannover, Foundation, Bünteweg 2, 30559, Hannover, Germany

**Keywords:** Dog, Sperm storage, Oviduct, Oocyte maturation, Fertilization

## Abstract

**Background:**

Unlike other domestic mammals, in which metaphase-II oocytes are ovulated, canine ovulation is characterized by the release of primary oocytes, which may take 12 to up to 36 hours. Further 60 hours are needed for maturation to secondary oocytes which then remain fertile for about 48 hours. Oestrus takes 7 to 10 days on average and may start as early as a week before ovulation. This together with the prolonged process of post-ovulatory oocyte maturation requires an according longevity of spermatozoa in the female genital tract in order to provide a population of fertile sperm when oocytes have matured to fertilizability. Therefore the distribution and viability of spermatozoa in the bitch genital tract was examined during post-ovulatory oocyte maturation.

**Methods:**

Thirteen beagle bitches were inseminated on the day of sonographically verified ovulation with pooled semen of two beagle dogs containing one billion progressively motile spermatozoa. Ovariohysterectomy was performed two days later (group 1, n = 6) and four days later (group 2, n = 7). The oviduct and uterine horn of one side were flushed separately and the flushing’s were checked for the presence of gametes. The oviducts including the utero-tubal junction and the uterine horns, both the flushed and unflushed, were histologically examined for sperm distribution.

**Results:**

The total number of spermatozoa recovered by flushing was low and evaluation of viability was limited. Prophase-I oocytes were collected from oviduct flushing in group 1, whereas unfertilized metaphase-II oocytes were detected in group 2. From day 2 to day 4 after ovulation a significant decrease in the percentage of glands containing sperm (P<0.05) and a marked reduction of the mean sperm number in uterine horn glands were observed. A concomitant diminution of spermatozoa was indicated in the utero-tubal junction accompanied by a slight increase in sperm numbers in the mid oviduct.

**Conclusions:**

Oocyte maturation to metaphase-II stage is accompanied by a continuous sperm detachment and elimination in the uterine horns. Entrance of spermatozoa into the caudal oviduct seems to be steadily controlled by the utero-tubal junction thus providing a selected sperm population to be shifted towards the site of fertilization when oocyte maturation is completed.

## Background

In most domestic mammals, the utero-tubal junction and the caudal isthmus have been demonstrated to build a functional sperm reservoir. Relative to the time interval from onset of oestrus to ovulation a population of viable spermatozoa must be able to survive in the female genital tract in cattle and sheep for at least 24 hours [1-4] and in swine for at least 40 hours [5]. Spermatozoa competent to penetrate an oocyte are suggested to be largely sequestered before ovulation in the caudal region of the oviduct possibly for 18 to 20 hours or more in cows [4], for 17 to 18 hours in ewes [3], and for at least 36 hours in sows [5]. Canine spermatozoa may remain motile [6] and even fertile for up to 11 days in the female genital tract [7]. There is evidence that canine sperm are stored in the utero-tubal junction and the uterine glands [8,9]. Only a few sperm have been detected in the oviductal isthmus, indicating that it is unlikely to be a storage site in the bitch [6,8-10]. Nevertheless, in *in vitro* preparations, spermatozoa were attached to the oviductal epithelium for 5 to 6 days [11].

In the dog primary oocytes are released from the ovaries over a period of 12 to 24 hours [12,13] and up to 36 hours [14]. Sixty hours are needed for maturation to secondary oocytes which then remain fertile for 48 hours [15]. However, despite the presence of both metaphase-II oocytes and spermatozoa for several hours, fertilization may be delayed at least up to 83 hours after ovulation, suggesting the need for a minimum period in the oviduct before fertilization [16,17].

Endocrine peculiarities of the bitch include the pre-ovulatory progesterone rise caused by follicular wall luteinisation as a consequence of the LH-surge. Corresponding progesterone concentrations in peripheral blood are 6.4-12.7 nmol/L [18-20], and 12.7-31.8 nmol/L at ovulation [18,21-26]. Consequently, post-ovulatory oocyte maturation is accompanied by a sharp progesterone rise, and fertilization usually takes place at progesterone concentrations above 47.7 nmol/L [27].

Based on the above mentioned dog specific features we hypothesized that following insemination shortly after ovulation a selected sperm population is provided in the cranial genital tract throughout post-ovulatory oocyte maturation for fertilization. Accordingly, the aim of the present study was to investigate the distribution and viability of spermatozoa in the genital tract of bitches in relation to oocyte maturation.

## Methods

### Animals

Thirteen clinically healthy beagle bitches (age: 14-47 months; body weight: 10.2-13.4 kg) and two healthy male beagles (each aged 28 months; body weight: 17.5 and 19.4 kg) were used in this study. The dogs were housed separated by gender in groups of two to four and were fed once daily with a commercial diet. Water was available *ad libitum*.

Animal housing, care and experimentation complied with the animal welfare regulations in Germany (Lower Saxony State Office for Consumer Protection and Food Safety, Approval Number 42502/05-10.05).

### Determination of ovulation

**Figure 1:**
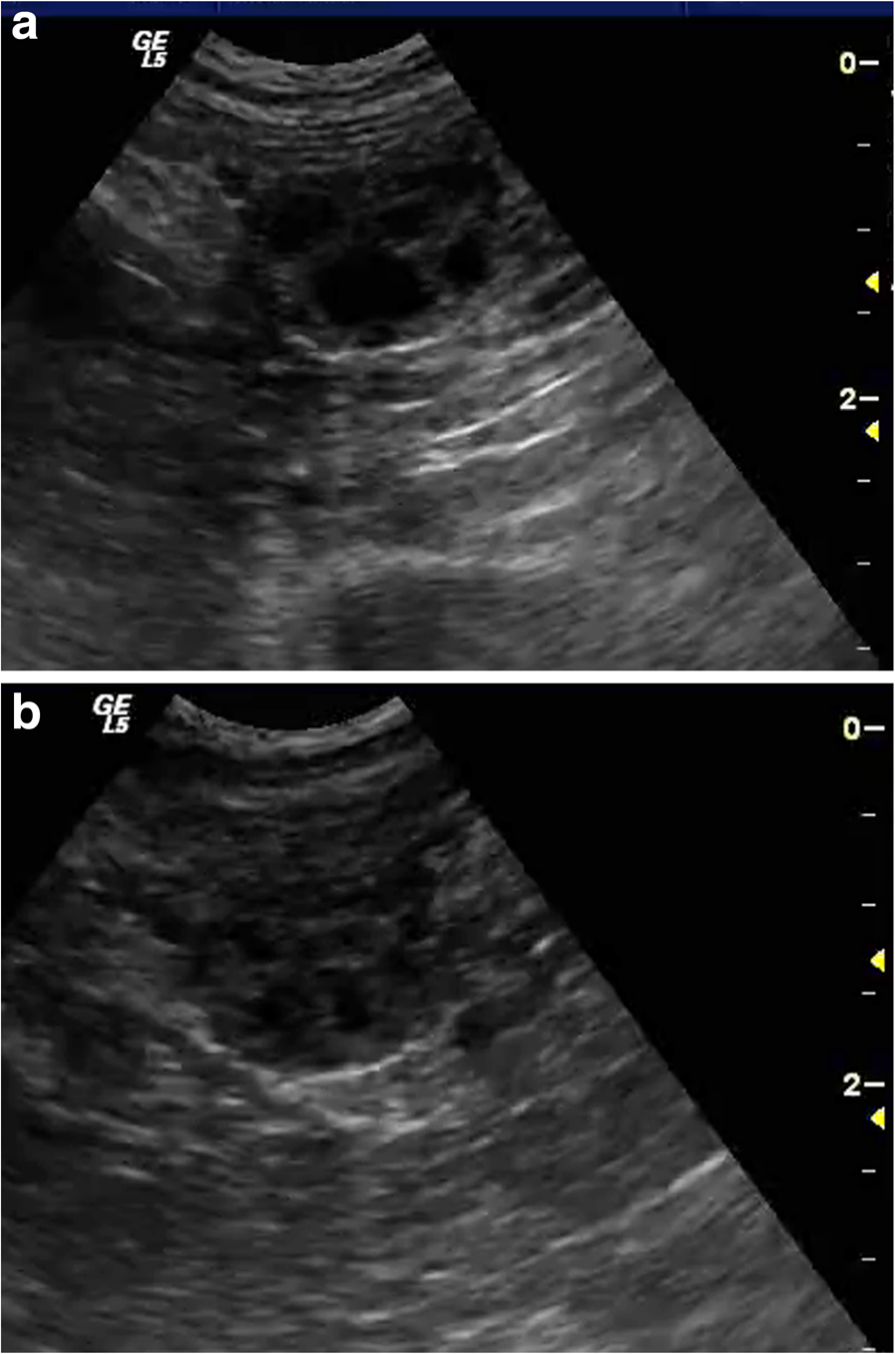
**Left ovary of the same bitch 24 hours before ovulation (a) and at ovulation (b).** The preovulatory follicles (**a**) are characterized by increasing echogenicity of the follicular wall indicating preovulatory luteinisation. Ovulation (**b**) is indicated by a significant decrease in the amount of fluid in the majority of follicular cavities.

The bitches were examined twice weekly for signs of proestrus. At first detection of proestrous bleeding, vaginoscopy and vaginal cytology were performed and repeated at two day intervals. Additional blood samples were taken for progesterone analysis in order to detect the initial pre-ovulatory progesterone rise. At a progesterone level of 6.4-9.5 nmol/L, ultrasonographic examination of the ovaries was started using a 10-MHz convex probe (Logiq 5 Pro, GE Medical Systems, Solingen, Germany), and repeated every 12 hours. For verification of ovulation the diameter of the largest follicle was measured at each examination and the serum progesterone concentration was determined once daily. Regarding the pre-ovulatory progesterone rise the reference to ovulation was set at a progesterone concentration of at least 15.9 nmol/L. As the process of ovulation may last for 12-36 hours [12-14] the mean time between the last ultrasound image with the majority of follicles visible (Figure 1a) and the ultrasound finding indicating disappearance of the majority or all follicles (Figure 1b) as described by other authors [14,28-30] was additionally used for determining ovulation.

### Progesterone analysis

Blood samples were centrifuged for 10 min at 3000 *g* for separation of blood serum. For progesterone analysis, a competitive enzyme immunoassay (Immulite, Siemens Diagnostics; detection limit 0.16 nmol/L, inter-assay-variation 12%, intra-assay-variation 4.6%) was used.

### Semen collection and preparation of the inseminate

Ejaculates were collected from the two semen donors by digital manipulation in the presence of a teaser bitch. The sperm rich fractions were examined for volume (ml), appearance (consistency, color), pH-value, sperm concentration (10^6^/ml), total sperm number (10^6^), sperm motility and morphology [31].

The insemination dosage of one billion progressively motile spermatozoa (total sperm number 1.05 to 1.3 billion) was prepared by mixing the sperm rich fractions of both dogs in roughly equal proportions. The resulting sperm volume was filled up with prostatic secretion from each ejaculate to a standardized volume of 3 ml. After aspiration of 3 ml of air, the insemination dosage was drawn into a sterile 6 ml syringe with Luer cone (B. Braun).

### Artificial insemination

A single intravaginal insemination was performed within 12 hours after detection of ovulation by means of a balloon catheter (Osiris Catheter, IMV Technologies) [32]. After insemination the catheter was left for a further 30 minutes in the vagina in order to mimic the duration of the genital lock during natural mating and by this provide sufficient time for the sperm to pass through the cervix. Furthermore the perineal region was gently stroked to stimulate uterine contractions and sperm transport.

### Ovariohysterectomy

Ovariohysterectomy was performed under general anesthesia 2 days (group 1, n = 6) and 4 days (group 2, n = 7) after ovulation. Before excision both uterine horns were ligated at the transition to the utero-tubal junction 1 cm caudal to the ovarian bursa to avoid artifacts in sperm distribution between the oviduct including the utero-tubal junction and the cranial uterine horn. A second ligation was fixed at the entrance into the uterine body to prevent sperm loss.

### Flushing

Immediately after excision, the genital tract was cut at the ligation sites and the oviducts with the appending utero-tubal junction were carefully separated from the ovarian bursa. Alternating from bitch to bitch the left or right oviduct and uterine horn were flushed separately using 5 ml Tyrode medium per flushing. Flushing was performed three times on the oviducts and once on the uterine horns. Then all genital tract segments (entire oviduct and utero-tubal junction, cranial and caudal uterine horn) of the flushed and unflushed side were fixed straight on Styrofoam plates using pins and transferred into 4% buffered formalin solution. The flushing’s were examined for the presence of spermatozoa and oocytes using a stereo microscope (Carl Zeiss). Oocytes were transferred into Eppendorf tubes with Karnowsky-liquid and stored at 4°C.

The flushing liquid was then centrifuged by 758 *g* at 25°C for 5 minutes, and the supernatant was removed and reduced to 2 ml. After repeated centrifugation, 1 ml of the supernatant was removed and the remaining fluid mixed by careful shaking. A total of ten 50 μl-drops were scanned on a slide under a cover glass and the sperm cells counted under a light microscope.

Oocytes were stained with Kamin-acetic acid (Division Chroma) for 5 minutes and rinsed with distilled water. After an increasing alcohol gradient and overnight evaporation of the residual alcohol in a liquid consisting of 100 ml Technovit 7100 and 1 g hardener I (Heraeus Kulzer) oocytes were transferred in 15 ml of liquid (Technovit® 7100 and 1 ml hardener II) into a Teflon form. From the resulting blocks 2 μm sections were cut by Auto cut (Reichert Jung), glued on slides at 90°C for 2 hours and then stained 1 minute in toluidine-blue (Merck). After rinsing in distilled water, oocyte sections were washed three times for 1 minute in 80% alcohol and twice for 1 minute in absolute alcohol. Then they were dried, covered by DePex mounting medium (Serva) and evaluated at magnification 400x using a BX60 microscope (Olympus).

### Histology

All genital tract segments were embedded in paraffin within one week after resection. Oviducts were divided into infundibulum, mid oviduct, caudal oviduct, and utero-tubal junction. Sections of 2 μm (10 from each oviductal location and 30 from each uterine location) were cut and mounted on slides coated with chromalaun-gelatine (Engelbrecht) and dried at 70°C. Considering the dimensions of the canine sperm head of 5 x 7 μm, a minimum of 10 μm of tissue was discarded to prevent double counting of spermatozoa.

Except for the utero-tubal junction, which was cut in longitudinal direction, all segments were cut in transverse section. The deparaffinised preparations were stained with hematoxylin-eosin (HE) and a cover slip was fixed using Roti-Histokitt (Carl Roth GmbH) [33].

Tissue sections were examined by light microscopy (160x/400x). For the oviduct and the uterine lumen, absolute sperm numbers were determined. Intra-glandular spermatozoa were recorded semi-quantitatively by classifying 100 glands per uterine section in glands with 0, 1, 2 to 5 and more than 5 spermatozoa.

### Statistical analysis

Data were analyzed by the statistic software SAS (Version 9.1, SAS Inc.). For statistical analysis uterine glands with 2-5 sperm and more than 5 sperm were defined as glands with 3.5 sperm and 6 sperm, respectively. WilcoxonÂ´s signed rank test for independent samples was used to compare (1) the sperm distribution in the genital segments on day 2 and day 4 after ovulation, (2) the number of spermatozoa in flushed and unflushed oviducts and uterine horns, and (3) the number of spermatozoa in left and right oviducts and uterine horns. P < 0.05 was considered significant. Due to the low sperm numbers collected by flushing, statistical analysis regarding motile spermatozoa was impossible.

## Results

### Criteria of ovulation and semen quality

The mean diameter of the largest follicle 12 hours prior to ovulation was 5.9 mm (5.0-8.6 mm). In 10 of the 13 bitches ovulation was sonographically characterized by a homogenous hypo echoic appearance of both ovaries indicating release of fluid from all follicular cavities. In the remaining three dogs follicles had completely disappeared in only one ovary, while in the other one remnants of fluid were visible in single follicular cavities. Ovulation occurred on average on day 11 (range day 9-14) after the onset of proestrus, at an overall mean progesterone concentration of 26.1 nmol/L (group 1: 30.5 nmol/L, range 15.9-41.0 nmol/L; group 2: 25.8 nmol/l, range 24.2-28.9 nmol/l). On the day of ovariohysterectomy, the mean progesterone concentration had increased to 67.7 nmol/L (38.2-84.0 nmol/L) in group 1 and to 93.8 nmol/L (77.0-111.3 nmol/L) in group 2.

The sperm rich fractions collected from the two semen donors and pooled had a mean total sperm number of 1237.3 x 10^6^ (572-2002 x 10^6^). The mean percentage of progressively motile spermatozoa was 86.9% (75-95%). On average 89.6% (80.5-98.0%) of spermatozoa had normal morphology and 88.5% (81-95%) had an intact plasma membrane.

### Spermatozoa and oocytes collected by flushing

Spermatozoa were found in the oviduct flushing’s in three bitches of group 1 (50%; bitches no. 1, 3, 6) and in four bitches of group 2 (57.1%; bitches no. 7, 10, 12, 13).

The number of bitches with spermatozoa detected in the uterine horn flushing’s was the same but individual bitches were partly differing (group 1: bitches no. 1, 3, 5; group 2: bitches no. 10, 11, 12, 13). In the samples obtained from bitch no. 3 (group 1) sperm counting and motility assessment was not possible due to agglutination of spermatozoa with erythrocytes.

The maximum sperm number obtained from the oviduct was 55 in group 1 and 73 in group 2 with 25% and 60% motile spermatozoa (progressive and local), respectively. The maximum sperm number collected from the uterine horn was 38 in group 1 and 138 in group 2 with 60% and 80% motile spermatozoa, respectively.

**Figure 2:**
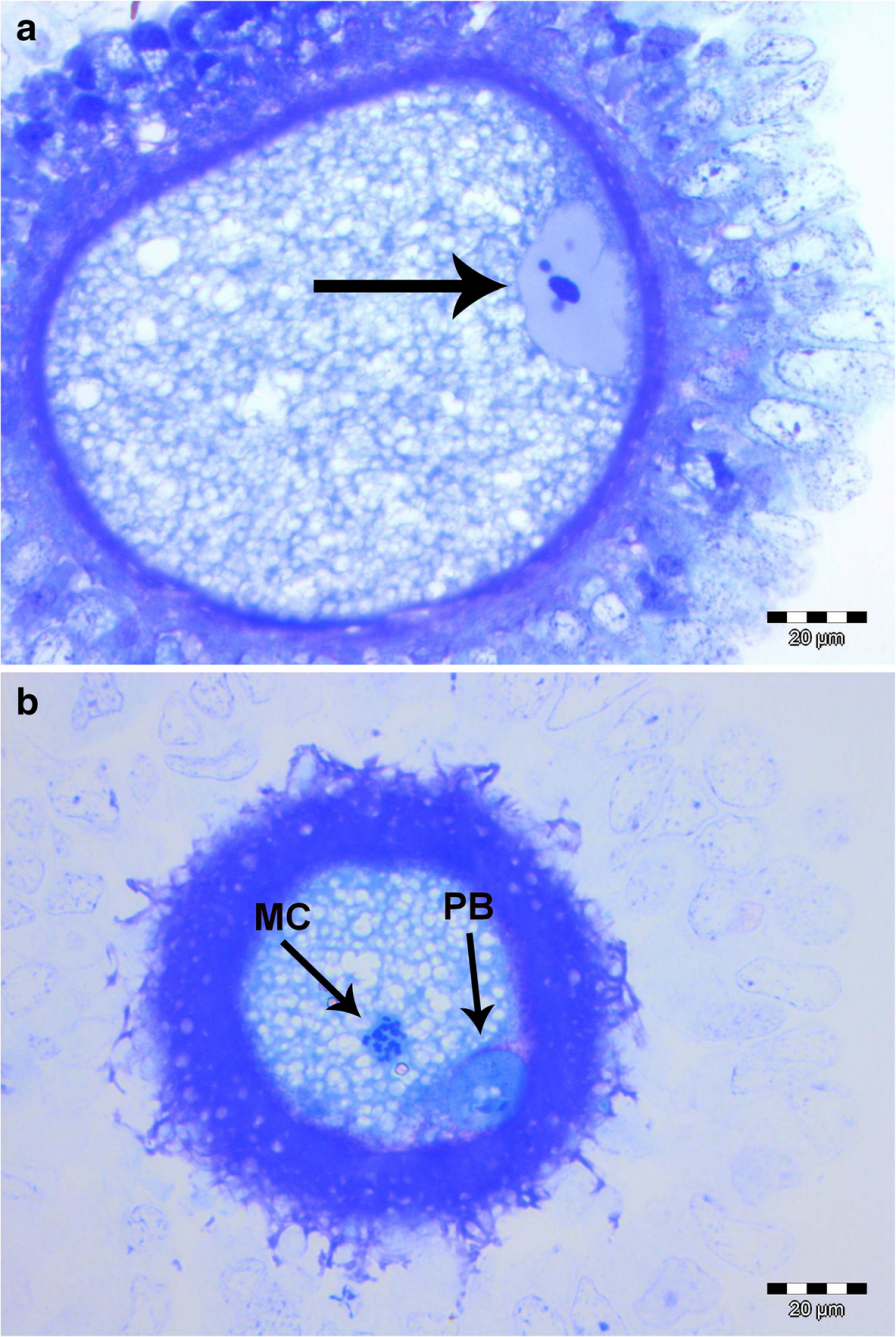
**Oocytes collected by oviduct flushing a. 2 days after ovulation/insemination presenting a peripherally located germinal vesicle (arrow) with an undulating nuclear envelope, b. in metaphase-II stage 4 days after ovulation/insemination.** The first polar body (PB) is extruded and the maternal chromatin (MC) can be recognized in the ooplasm.

A total of 53 oocytes were flushed from the unilateral oviducts of 12 out of 13 bitches (92.3%), 39 of which were evaluated for maturation status. All eleven oocytes collected 2 days after ovulation were in the prophase-I stage (Figure 2a), whereas 27 out of 28 oocytes collected 4 days after ovulation had reached the metaphase-II stage (Figure 2b). No signs of fertilization were detected.

### Histological evaluation of sperm distribution in the uterus and oviduct

The total sperm number in the uterine horn and oviduct segments varied considerably between individual bitches of each group (Tables 1 and 2), resulting in the lack of statistical significances. The same was true regarding the sperm number in the flushed and unflushed oviducts [(two days after ovulation: flushed – mean 56.5 (0-169), unflushed – mean 265.0 (0-1558); four days after ovulation: flushed – mean 1.5 (0-3), unflushed – mean 15.9 (0-51)].

**Table 1:** Mean number of glandular and luminal spermatozoa in the cranial and caudal uterine horn (UH) segments of Beagle bitches 2 and 4 days after ovulation and vaginal insemination of one billion progressively motile spermatozoa

	**Two days after ovulation (*n*** **= 6)**			**Four days after ovulation (*n*** **= 7)**		
	**Left**	**Right**	**Total**	**Left**	**Right**	**Total**
**Cranial UH**						
Glands						
Mean	995.6	759.5	1755.1	197.3	172.0	369.3
± SD	1448.7	1073.6	2521.7	231.4	244.0	432.8
Range	0-1252.5	0-946	0-5820	0-242	0-245.5	0-1136
Lumen						
Mean	198.7	441.7	640.4	5.4	4.4	9.8
± SD	319.4	761.4	1078.1	7.8	7.3	10.8
Range	0-541	0-1724	0-1854	0-15	0-16	0-25
**Caudal UH**						
Glands						
Mean	978.2	787.2	1765.4	161.8	203.5	365.3
± SD	1279.7	1030.7	2305.3	207.9	334.3	527.7
Range	0-1065	0-806	0-5066	0-205.5	0-350.5	0-1444.5
Lumen						
Mean	447.2	197.2	644.4	3.9	3.9	7.8
± SD	1021.7	350.9	1361.6	5.7	8.1	12.9
Range	0-1556	0-456	0-1896	0-12	0-13	0-36

**Table 2:** Mean number of spermatozoa in the oviductal segments of Beagle bitches 2 and 4 days after ovulation and vaginal insemination of one billion progressively motile spermatozoa

	**Two days after ovulation (*n*** **= 6)**			**Four days after ovulation (*n*** **= 7)**		
	**Left**	**Right**	**Total**	**Left**	**Right**	**Total**
Utero-tubal junction						
Mean ± SD	5.0 ±6.3	313.2 ±614.9	318.2 ±614.8	4.0 ±8.5	12.0 ±20.4	16.0 ± 19.8
Range	0-14	0-1558	0-1561	0-23	0-51	0-51
Caudal oviduct						
Mean ± SD	2.0 ±3.6	0.0	2.0 ±3.6	0.6 ±1.1	0.7 ±1.5	1.3 ±2.0
Range	0-9	0	0-9	0-3	0-4	0-5
Mid oviduct						
Mean ± SD	0.2 ±0.4	0.2 ±0.4	0.3 ±0.5	0.4 ±1.1	1.7 ±2.9	2.1 ±2.8
Range	0-1	0-1	0-1	0-3	0-8	0-8
Infundibulum						
Mean ± SD	0.7 ±0.8	0.3 ±0.8	1.0 ±1.3	0.0	0.1 ±0.4	0.1 ±0.4
Range	0-2	0-2	0-3	0	0-1	0-1

In both oviducts collected from a bitch day 2 after ovulation no spermatozoa were found (see also Table 1, bitch no. 5).

**Figure 3:**
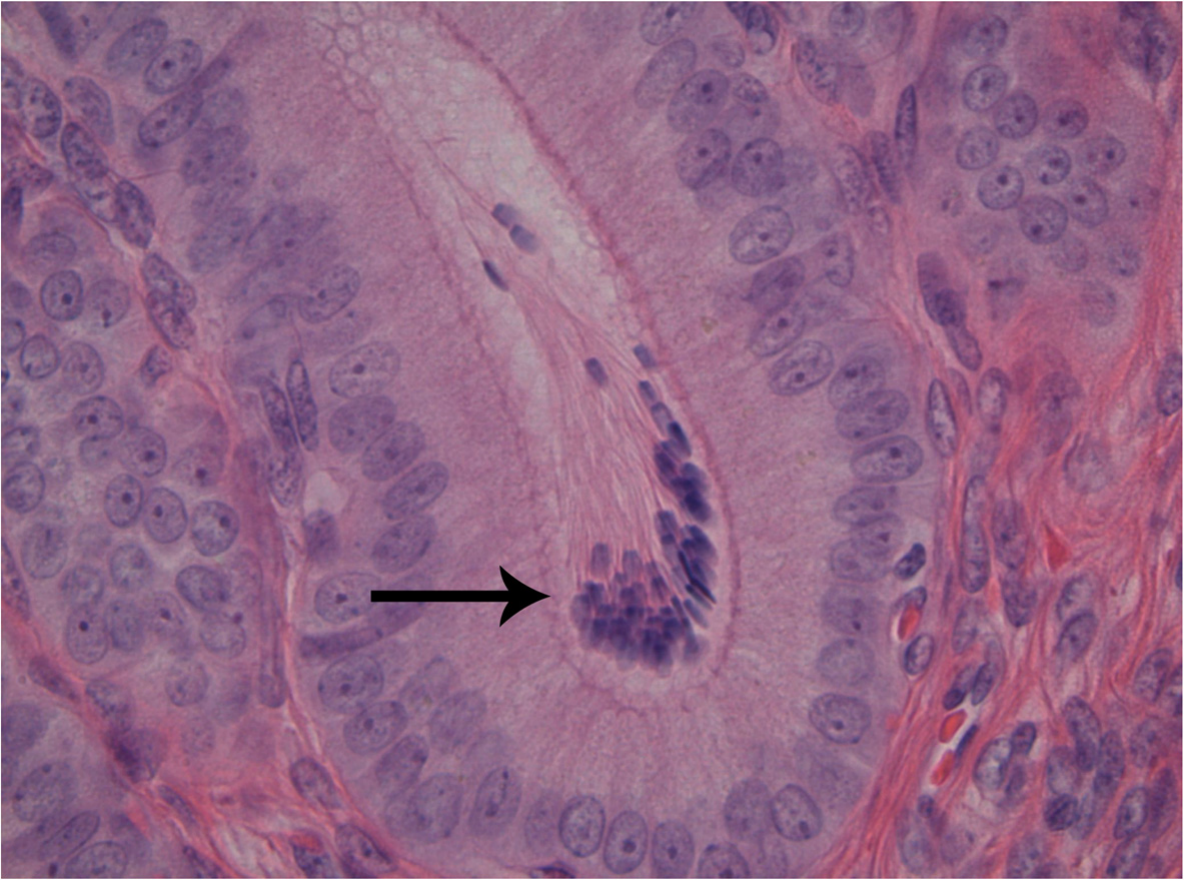
Accumulation of spermatozoa (arrow) in a uterine horn gland.

The mean percentages of uterine glands containing sperm were 7.1% in group 1 and 2.0% in group 2 (P<0.05). Glands with more than 5 spermatozoa (Figure 3) were found more frequently 2 days after ovulation (49.8%) than 4 days after ovulation (23.3%), the difference showing no statistical significance. Sperm distribution was almost identical in the glands and lumen of the caudal and cranial uterine horn segments. On day 2 and day 4 the mean total number of glandular sperm in the uterine horns was 3520.5 and 734.6, respectively. The mean total number of luminal sperm was 1284.8 on day 2 and 17.6 on day 4 after ovulation.

**Figure 4:**
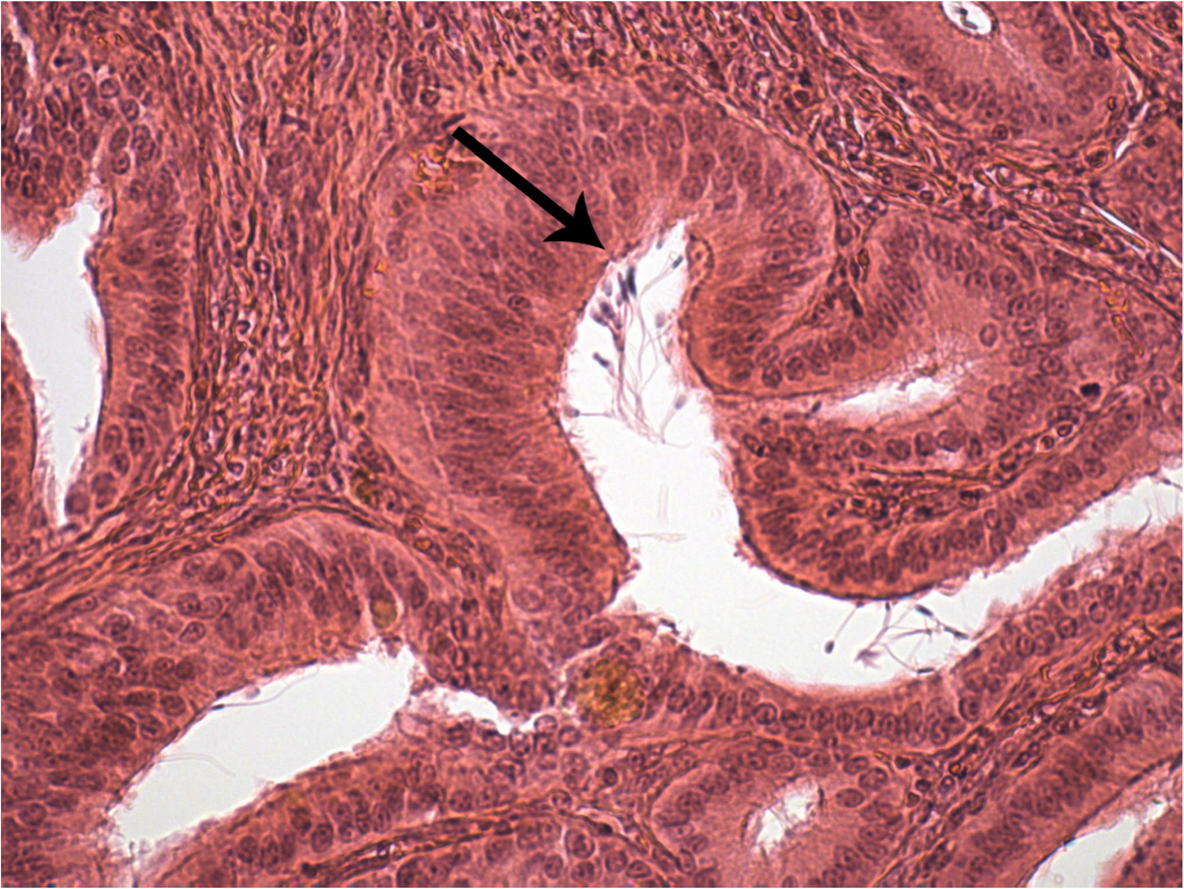
**Spermatozoa (arrow) in the grooves formed by folds of the mucosal epithelium of the utero-tubal junction.** The majority of cells seem to be attached to the epithelium.

Out of the oviductal segments the utero-tubal junction showed the largest amount of spermatozoa both, 2 and 4 days after ovulation (Figure 4), with a slight decrease in between (Table 2). The mean sperm number in the caudal oviduct appeared to be about stable whereas in the mid oviduct a slight increase was recognizable (Table 2).

## Discussion

In the present study particular importance was addressed to precise classification of the post-ovulatory period regarding sperm distribution and oocyte maturation. For this, ovulation was determined by real-time ultrasound examination combined with progesterone analysis. Pre-ovulatory follicle diameters matched with the data described by other authors [28,34,35]. The mean progesterone concentration of 26.1 nmol/L measured at the time of sonographically indicated ovulation was within the average range of 12.7-31.8 nmol/L described in the literature (see introduction). Variation of progesterone values (15.9-41.0 nmol/L) between bitches was probably due to inconsistency in pre-ovulatory follicle luteinisation [36] as well as to the variability in the time span between the pre-ovulatory LH-surge and ovulation, which has been shown to range from 24 to more than 96 hours [19]. Furthermore, differences in ovulatory progesterone concentrations between studies may partly result from different assay systems. The reliability of our methods applied for diagnosing ovulation is verified indirectly by the maturation stages of oocytes recovered 2 and 4 days later. On day 2 all oocytes were still in prophase-I stage, which has been shown to exist for up to 48 hours after ovulation [15,16], whereas on day 4 metaphase-II oocytes, which can be expected the earliest 48 to 54 hours after ovulation [15,16], were identified. Despite progesterone concentrations clearly above 47.7 nmol/L, as described by Concannon et al. (1975) [27] at the time of fertilization, signs of fertilization were missing probably due to a delay observed by Reynaud et al. (2005) [16] at least up to 83 hours after ovulation resulting in fertilization the earliest 90 hours after ovulation.

In accordance with previous reports [8,9] our results show that the uterine glands are a significant site of sperm storage in the dog which may, however, act primarily as sperm barriers by retaining a specific population of spermatozoa. By this the uterine glands may play an important role as selection mechanism for a viable sperm population, which is provided in the glandular region of the utero-tubal junction throughout the protracted time of post-ovulatory oocyte maturation and fertilization [8,9].

In the cat, a species with coitus induced ovulation of fertilizable metaphase-II oocytes, the uterine crypts and the utero-tubal junction have been shown to serve as sperm reservoirs prior to ovulation whereas the oviductal isthmus is suggested to be a site of sperm storage close to the time of ovulation and fertilization [37].

Sperm binding to the reproductive epithelium is the crucial mechanism for slowing destabilization of sperm membranes as part of the capacitation process [38,39] and allowing the timely maturation of sperm in relation to physiological stimuli from the bitch. The latter may include a rise in progesterone concentrations and factors deriving from the maturing oocytes [10,40,41]. In our study despite the low sperm numbers detected in oviduct flushing, viable sperm were found both 2 days and 4 days after ovulation with a maximum proportion of 60% motile spermatozoa (group 2) similar to the *in vitro* findings of England and Pacey (1998) [42] and Pacey et al. (2000) [43].

Two days and 4 days after ovulation, progesterone concentrations had reached high mean levels of 67.7 nmol/L and 93.8 nmol/L, respectively. In this period spermatozoa within the uterine horns were gradually eliminated, as indicated by decreasing absolute sperm numbers (Table 1) as well as by the shift from glands containing more than 5 sperm to glands containing less than 5 or even no sperm, also described by Rijsselaere et al. (2004) [8].

Elimination of spermatozoa from the genital tract has also been observed in the progesterone-influenced post-ovulatory period of the cat, 48 and 96 hours after mating corresponding to approximately 16 to 23 hours and 64 to 71 hours after ovulation, respectively [37]. It is suggested that in the bitch spermatozoa detaching from the uterine glands due to the increased progesterone concentrations may be eliminated from the genital tract whereas at least some of the sperm released at the same time from the utero-tubal junction may reach the site of fertilization.

In our study the reduction of sperm number in the utero-tubal junction was similar to that observed by Rijsselaere et al. (2004) [8] in four and three bitches, respectively, from day 1 to day 3 to 4 after ovulation, although all seven bitches had been inseminated only 24 hours prior to ovariohysterectomy, indicating that during this period the rate of both sperm binding and detachment may depend primarily on the concomitant progesterone rise and oocyte maturation rather than on the time of insemination.

Sperm detachment in the utero-tubal junction from day 2 to day 4 after ovulation (Table 1 and 2) was accompanied by a slight decrease of sperm numbers in the caudal oviduct. This finding may be supported by Urhausen et al. (2011) [44], who found a significant increase of apoptotic cells in the epithelium of the utero-tubal junction and caudal oviduct from day 2 to day 4 after ovulation, suggesting apoptosis to be an underlying mechanism of sperm detachment. The concomitant slight increase of the sperm number in the mid oviduct may provide evidence of a shift of spermatozoa to the site of fertilization. Redistribution of spermatozoa from the isthmus to the site of fertilization has also been shown to be influenced by ovulation and/or by mature oocytes in rabbits and pigs [45,46].

## Conclusions

The results of our study indicate that following vaginal insemination shortly after ovulation the majority of spermatozoa is retained in the uterine glands. A selected sperm population is stored in the utero-tubal junction and limited numbers of spermatozoa may be continuously released into the caudal oviduct until oocytes have matured to metaphase-II stage. Final oocyte maturation seems to be accompanied by a shift of spermatozoa from the caudal oviduct towards the site of fertilization.

## Competing interests

The authors declare that they have no competing interests.

## Authors’ contributions

IK realized the experimental part of the study including sonographic determination of ovulation, preparation of the genital tracts, flushing of oviducts and uterine horns, evaluation of sperm distribution in histological preparations and drafted the manuscript draft. AM-L performed the ovariohysterectomies under general anaesthesia. CU was involved in the conception and design of the study, in gynaecological examinations and collection of blood samples for determination of ovulation as well as in evaluation of flushings for the presence of oocytes and spermatozoa. AB was involved in the conception and design of the study, advised and supervised histological preparation and evaluation of tissue sections. BM supervised the collection and preparation of oocytes and evaluated the oocytes regarding maturation status. MP performed the progesterone analyses. MB was involved in the conception of the study, and performed the statistical analysis. AG-A had the main responsibility for the conception, design and realization of the study including the relevant aspects of animal welfare regulations, and revised the manuscript to its final version. All authors read and approved the final manuscript.
